# The challenge of diabetes treatment in Macedonia

**DOI:** 10.1186/1878-5085-5-S1-A68

**Published:** 2014-02-11

**Authors:** Ivica Smokovski

**Affiliations:** 1 Cabinet of Minister, Ministry of Health, Macedonia, FYROM

## 

Based on the International Diabetes Federation (IDF) estimates, there were 136.700 people with diabetes mellitus in Republic of Macedonia in 2012, and this number is projected to increase to 166.000 in 2030. National Guidelines on the treatment and control of type 2 diabetes have already been published in the Official Journal.

Most modern diabetes treatments are available; reimbursement covering test-strips metformin, sulphonylureas, meglitinides, human and analogue insulins, and certain number of insulin pumps. Insulins, analogue and human, are fully reimbursed, with no co-payment by the patients, and are fully covered by the State Budget for all patients, no matter if insured or not. More than half of the all treated diabetes patents are insulin treated, with 83% and 92% penetration of insulin analogues in volume and value, respectively, considerably higher relative to many more developed EU/EEA countries.

Macedonia appears to be among the European and world leaders if cost of insulin analogues as fraction of Gross Domestic Product per capita is taken into consideration, resulting in a challenge for the sustainability of the system since a major part of the diabetes care budget is allocated for insulin analogues. The system is facing a need to rationalize the insulin treatment costs given the expected steep rise in the diabetes prevalence, and the need of comprehensive diabetes care including emerging treatments.

